# Hepatic LDL receptor-related protein-1 deficiency alters mitochondrial dynamics through phosphatidylinositol 4,5-bisphosphate reduction

**DOI:** 10.1016/j.jbc.2021.100370

**Published:** 2021-02-03

**Authors:** Sivaprakasam Chinnarasu, Fawzi Alogaili, Kevin E. Bove, Anja Jaeschke, David Y. Hui

**Affiliations:** 1 Department of Pathology and Laboratory Medicine, Metabolic Diseases Research Center, University of Cincinnati College of Medicine, Cincinnati, Ohio, USA; 2 Department of Pathology and Laboratory Medicine, Cincinnati Children’s Hospital Medical Center, Cincinnati, Ohio, USA

**Keywords:** respiration, liver metabolism, lipoprotein receptor, lipoprotein receptor-related protein (LRP), cardiolipin, inositol phosphate, CDP-DAG, cytidine-5’ diphosphate-diacylglycerol, DAG, diacylglycerol, DRP1, dynamin-related protein-1, FCCP, carbonyl cyanide *p*-trifluoromethoxyphenylhydrazone, LRP1, low-density lipoprotein receptor-related protein-1, MFN2, mitofusin-2, OPA1, dynamin-like GTPase, PI(4,5)P_2_, phosphatidylinositol(4,5)bisphosphate, PIP5K, phosphatidylinositol-4-phosphate-5 kinase, PIP5Kl1, PIP5K like protein-1, PLCγ1, phospholipase C-γ1

## Abstract

The LDL receptor-related protein 1 (LRP1) is a multifunctional transmembrane protein with endocytosis and signal transduction functions. Previous studies have shown that hepatic LRP1 deficiency exacerbates diet-induced steatohepatitis and insulin resistance *via* mechanisms related to increased lysosome and mitochondria permeability and dysfunction. The current study examined the impact of LRP1 deficiency on mitochondrial function in the liver. Hepatocytes isolated from liver-specific LRP1 knockout (*hLrp1*^*−/−*^) mice showed reduced oxygen consumption compared with control mouse hepatocytes. The mitochondria in *hLrp1*^*−/−*^ mouse livers have an abnormal morphology and their membranes contain significantly less anionic phospholipids, including lower levels of phosphatidylethanolamine and cardiolipin that increase mitochondrial fission and impair fusion. Additional studies showed that LRP1 complexes with phosphatidylinositol 4-phosphate 5-kinase like protein-1 (PIP5KL1) and phosphatidylinositol 4-phosphate 5-kinase-1β (PIP5K1β). The absence of LRP1 reduces the levels of both PIP5KL1 and PIP5K1β in the plasma membrane and also lowers phosphatidylinositol(4,5) bisphosphate (PI(4,5)P_2_) levels in hepatocytes. These data indicate that LRP1 recruits PIP5KL1 and PIP5K1β to the plasma membrane for PI(4,5)P_2_ biosynthesis. The lack of LRP1 reduces lipid kinase expression, leading to lower PI(4,5)P_2_ levels, thereby decreasing the availability of this lipid metabolite in the cardiolipin biosynthesis pathway to cause cardiolipin reduction and the impairment in mitochondria homeostasis. Taken together, the current study identifies another signaling mechanism by which LRP1 regulates cell functions: binding and recruitment of PIP5KL1 and PIP5K1β to the membrane for PI(4,5)P_2_ synthesis. In addition, it highlights the importance of this mechanism for maintaining the integrity and functions of intracellular organelles.

Low-density lipoprotein receptor-related protein-1 (LRP1) is a type 1 transmembrane protein that serves endocytic and signaling functions through mechanisms that are cell type and context-dependent. For example, in macrophages, LRP1 is an endocytosis receptor for the uptake of aggregated LDL and efferocytosis of dead cells (1, 2). LRP1 in macrophages also serves important signal transduction functions including the activation of phosphatidylinositol 3-kinase to suppress toll-like receptor-induced inflammation (3), as well as the activation of liver X receptor to increase ABCA1 expression to limit cholesterol accumulation (4). The cardiometabolic benefits of macrophage LRP1 signaling are counterbalanced by its enhancement of Wnt signaling that leads to hepatic inflammation and insulin resistance (5). In smooth muscle cells, the role of LRP1 is primarily signal transduction regulation in limiting cellular response to platelet-derived growth factor and transforming growth factor-β (6). The lack of LRP1 in smooth muscle cells accelerates cholesterol-induced atherosclerosis as well as promotes injury-induced neointimal hyperplasia and vascular cardiomyopathy in a cholesterol-independent manner (7, 8, 9). In preadipocytes, LRP1 is an integrator of adipogenic differentiation and fat storage signals through activation of Wnt signaling pathway (10), and the absence of LRP1 impairs adipocyte differentiation (11, 12). In mature adipocytes, LRP1 is required for basal lipolysis and insulin-induced translocation of glucose transporter-4 to the plasma membrane (10, 13). The absence of LRP1 in mature adipocytes impairs lipid accumulation to limit diet-induced adiposity but also promotes inflammation in exacerbation of atherosclerosis (14, 15). In the brain, LRP1 participates in the production and clearance of amyloid-β peptide and limits neuroinflammation (16, 17, 18). LRP1 is also abundantly expressed in the liver, where it serves primarily an endocytic function and works in conjunction with LDL receptor for plasma clearance of apoE-containing lipoproteins (19).

Recent studies revealed that LRP1 also plays a regulatory role in hepatocytes. The absence of LRP1 in hepatocytes reduces cell surface expression of ABCA1, leading to the lowering of HDL level in the plasma of hepatocyte-specific LRP1 knockout (*hLrp1*^*−/−*^) mice (20). The *hLrp1*^*−/−*^ mice are also more sensitive to high fat diet–induced hepatosteatosis and insulin resistance (21), with accelerated progression to steatohepatitis when fed a high fat diet with additional cholesterol supplementation (22). The mechanism underlying the increased sensitivity of *hLrp1*^*−/−*^ mice to diet-induced steatosis and toxicity is attributed to increased lysosomal and mitochondrial permeability as a consequence of lipid overload (23). However, whether LRP1 deficiency directly influences the functions of these intracellular organelles in the absence of elevated lipid deposition remains to be clarified. The goal of this study is to examine the effect of LRP1 deficiency on mitochondrial functions in hepatocytes.

## Results

### LRP1 deficiency reduces mitochondrial respiration in hepatocytes

**Figure 1:**
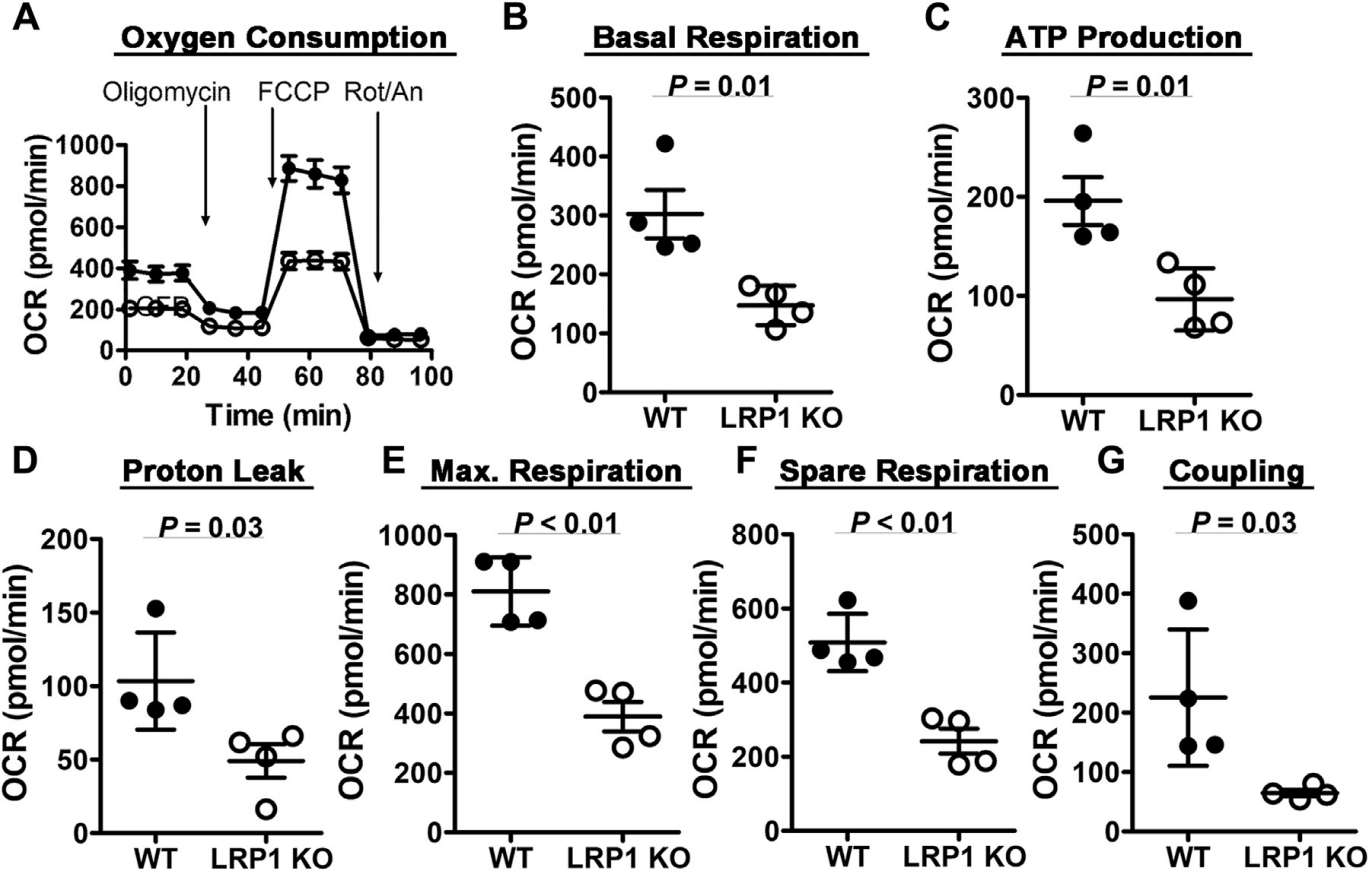
**Hepatic LRP1 deficiency reduces mitochondrial respiration and ATP production.***A*, primary hepatocytes isolated from wild-type (*solid symbols*, WT) or *hLrp1*^*−/−*^ mice (*open symbols*, LRP1 KO) (n = 4) were incubated in medium containing 25 mM glucose, 1 mM sodium pyruvate, and 4 mM Glutamax to determine basal oxygen consumption rates. Oligomycin, FCCP, and rotenone/antimycin were added to measure ATP production, maximum and spare respiration, and coupling efficiency. The data were used to determine basal respiration rate (*B*), ATP production (*C*), proton leak (*D*), maximum respiration rate (*E*), spare respiration (*F*), and coupling efficiency (*G*). The data are reported as mean ± SD and were evaluated by two-tailed Student’s *t*-test for statistical significance as indicated.

**Figure 2:**
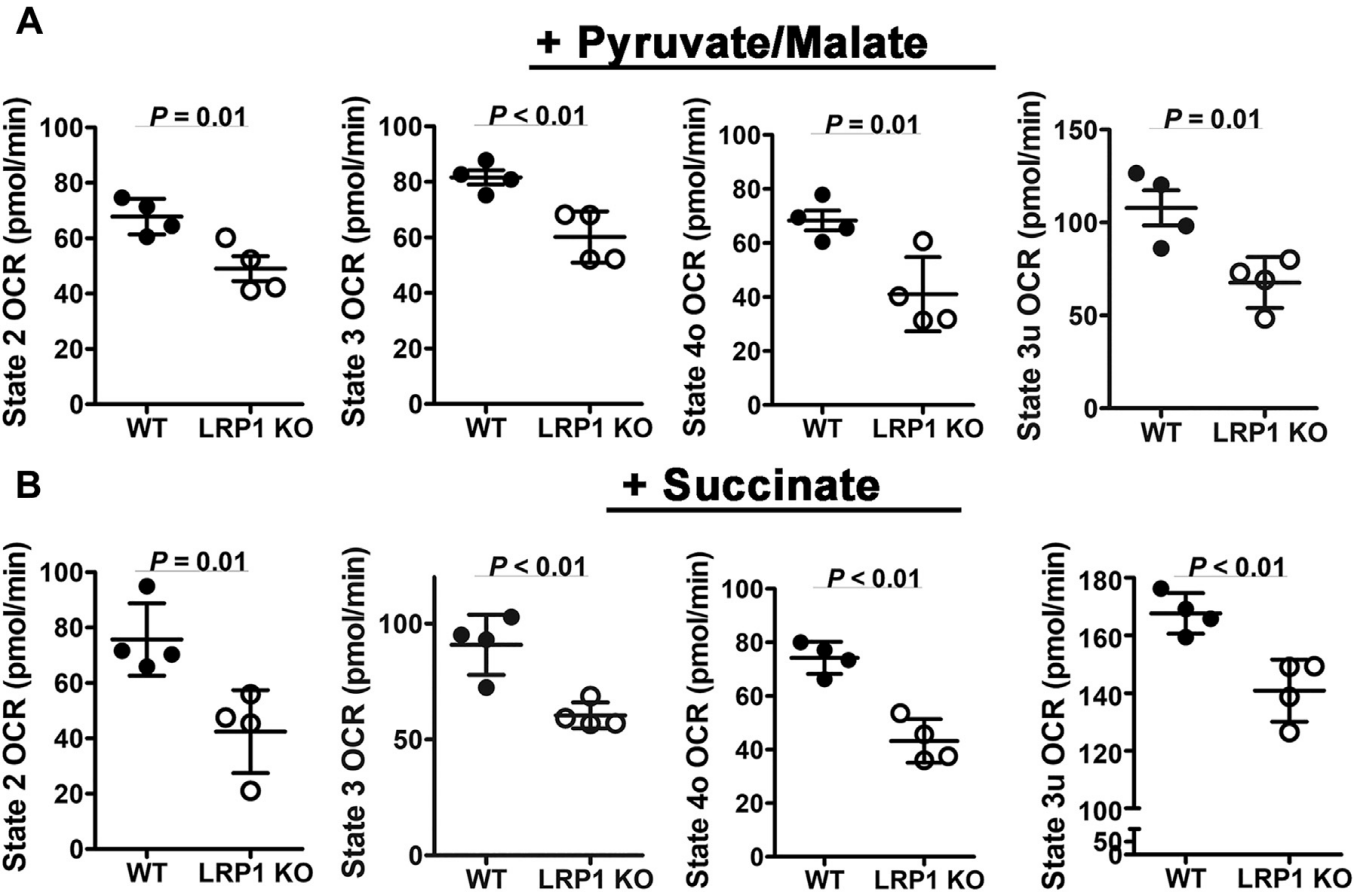
**Hepatic LRP1 deficiency reduces (*A*) pyruvate/malate- and (*B*) succinate-mediated mitochondrial respiration.** Primary hepatocytes isolated from wild-type (*solid bars*, WT) or *hLrp1*^*−/−*^ mice (*open bars*, LRP1 KO) (n = 4) were incubated in medium containing (*A*) pyruvate and malate or (*B*) succinate to determine basal oxygen consumption rates. State 2 respiration was assessed in the presence of the indicated substrates. State 3 respiration was assessed with ADP addition. State 4o respiration was assessed based on oxygen consumption in the presence of oligomycin, and state 3u respiration was assessed in the presence of FCCP. The data are reported as mean ± SD and were evaluated by two-tailed Student’s *t*-test with statistical significance as indicated.

**Figure 3:**
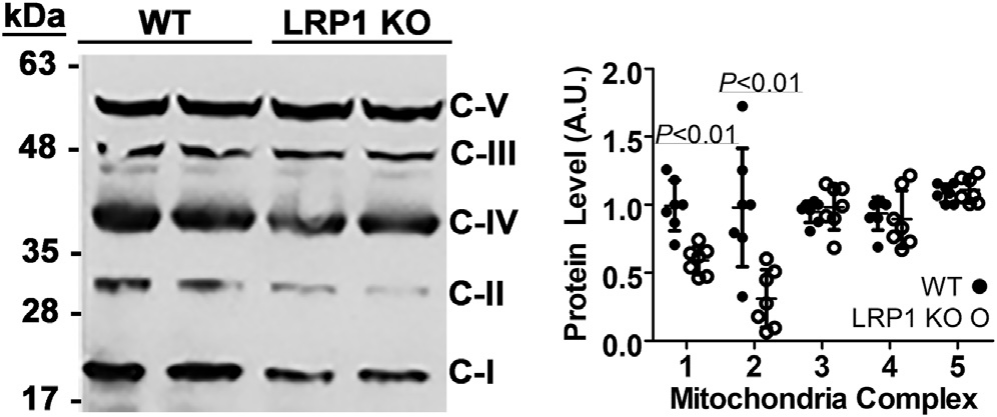
**Hepatic LRP1 deficiency reduces mitochondria complex I and complex II levels.** Liver lysates were prepared from wild-type (WT, *filled symbols*) and *hLrp1*^*−/−*^ (LRP1 KO, *open symbols*) mice for western blot analysis with antibody cocktail against rodent oxidative phosphorylation proteins. The *left panel* shows representative western blot image with molecular size markers and the identification of complex I through V (C-I through C-V) as indicated. The *right panel* shows the relative expression levels of each mitochondria complex in seven different liver preparations in each group. The data are reported as mean ± SD and were evaluated by two-tailed Student’s *t*-test for statistical significance as indicated.

Primary hepatocytes isolated from chow-fed C57BL/6J wild-type and *hLrp1*^*−/−*^ mice were incubated *in vitro* with medium containing 25 mM glucose to mimic the acute postprandial glycemic response *in vivo*. Oxygen consumption rates monitored over a 2-h period showed that the basal mitochondrial respiration rate was lower in *hLrp1*^*−/−*^ hepatocytes compared with wild-type cells (Fig. 1, *A* and *B*). The sequential addition of oligomycin, carbonyl cyanide *p*-trifluoromethoxyphenylhydrazone (FCCP), and then rotenone plus antimycin A during the 2-h incubation period revealed significant reduction in ATP production, proton leak, maximal and spare respiration rates, as well as lower coupling efficiency in hepatocytes with LRP1 deficiency (Fig. 1, *C*–*G*). Additionally, LRP1 inactivation was also found to lower oxygen consumption rates in response to pyruvate and malate- or succinate-driven respiration (Fig. 2, *A* and *B*). Analysis of the data revealed that LRP1 deficiency reduces oxygen consumption at all three stages of the citric acid cycle (Fig. 2, *A* and *B*). We interpret these data to indicate that LRP1 inactivation reduces respiration by lowering mitochondria complex I and complex II activities in hepatocytes. Western blot analysis of hepatocyte lysates prepared from wild-type and *hLrp1*^*−/−*^ mice with an antibody cocktail against rodent oxidative phosphorylation proteins confirmed the reduced levels of mitochondria complex-I and -II with LRP1 deficiency (Fig. 3).

### LRP1 deficiency increases mitochondria calcium uptake and accumulation with loss of membrane potential

**Figure 4:**
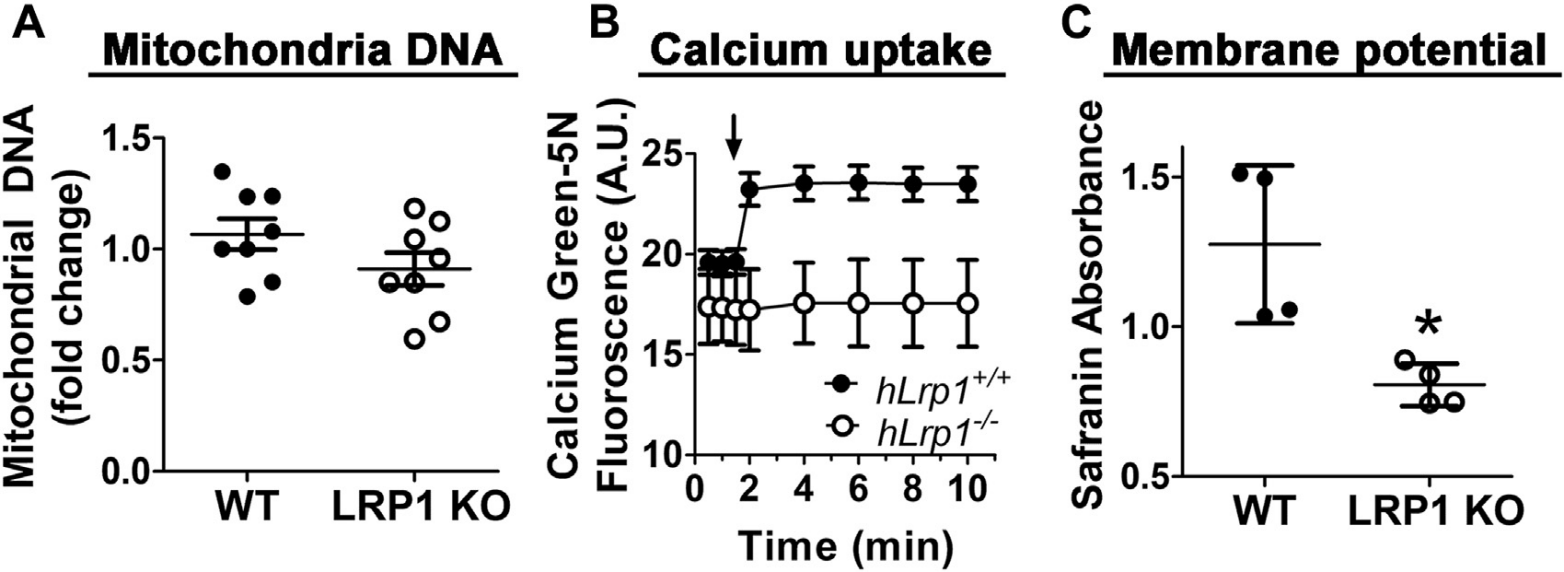
**LRP1 deficiency has no impact on mitochondria number but enhances calcium accumulation and induces membrane potential loss.***A*, mitochondria number was determined based on mitochondrial DNA content in the livers of eight wild-type (WT) and eight *hLrp1*^*−/−*^ (LRP1 KO) mice. *B*, mitochondria were isolated from WT (*solid symbols* and *bars*) and *hLrp1*^*−/−*^ (*open symbols* and *bars*) mice and incubated with the calcium indicator Calcium Green-5N prior to the addition of 75 μM CaCl_2_ at the time indicated by the *arrow*. Calcium levels in the medium were determined based on Calcium Green-5N fluorescence over time. *C*, membrane potential was assessed by absorbance measurement after incubation with the membrane voltage indicator safranin O. The data are reported as mean ± SD from four separate preparations and were evaluated by two-tailed Student’s *t*-test for statistical significance. ∗ indicates difference from wild type at *p* = 0.01.

The differences in mitochondrial respiration activities between wild-type and *hLrp1*^*−/−*^ hepatocytes are not due to differences in the number of mitochondria, as noted by comparable mitochondrial DNA content in wild-type and *hLrp1*^*−/−*^ mouse livers (Fig. 4*A*). These results suggest that LRP1 deficiency directly impacts mitochondrial functions in hepatocytes. This hypothesis was tested by comparing calcium uptake and accumulation by mitochondria isolated from the livers of wild-type and *hLrp1*^*−/−*^ mice. In these experiments, the isolated mitochondria were suspended in buffer containing Calcium Green-5N, a membrane-impermeable fluorescent calcium indicator, prior to the addition of 75 μM CaCl_2_. The addition of calcium to wild-type mitochondria incubation resulted in rapid increase in Calcium Green-5N fluorescence, indicative of elevated Ca^2+^ levels in the medium and the slow removal of calcium from the buffer by wild-type mitochondria (24). In contrast, minimal fluorescence increase was observed when calcium was added to mitochondria isolated from *hLrp1*^*−/−*^ mice (Fig. 4*B*). These data indicated the rapid accumulation of Ca^2+^ in mitochondria isolated from *hLrp1*^*−/−*^ mouse livers compared with that observed with wild-type mouse livers (Fig. 4*B*). The increased calcium uptake of mitochondria from *hLrp1*^*−/−*^ mice was reflected by their lower membrane potential compared with mitochondria obtained from the livers of wild-type mice (Fig. 4*C*).

### LRP1 deficiency alters mitochondrial membrane phospholipids and morphology

**Figure 5:**
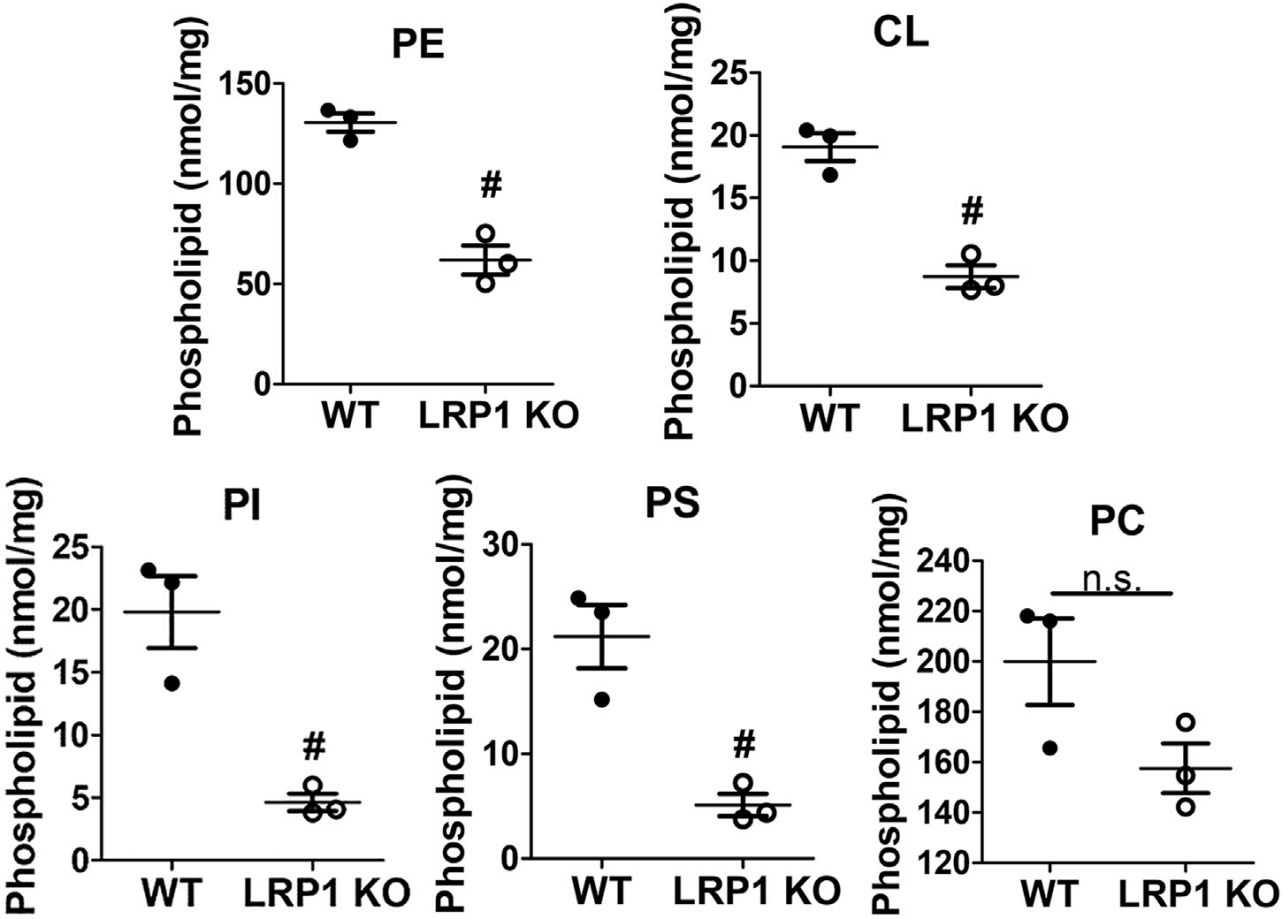
**Hepatic LRP1 deficiency reduces anionic phospholipid content in the mitochondria.** Lipids in the mitochondria isolated from wild-type (*solid bars*) and *hLrp1*^*−/−*^ (*open bars*) mouse livers were extracted with chloroform:methanol:water (2:1:3, v/v/v) and then applied to thin layer chromatography plates for phospholipid separation. Migration of the phospholipids was compared with standards. Phospholipid spots on the chromatography plates corresponding to phosphatidylethanolamine (PE), cardiolipin (CL), phosphatidylinositol (PI), phosphatidylserine (PS), and phosphatidylcholine (PC) were scraped and quantified by phosphorus measurements. The data are reported as mean ± SD from N = 3 mice in each group. The data were evaluated by two-tailed Student’s *t*-test for statistical significance. # indicates difference from wild type at *p* < 0.01.

**Figure 6:**
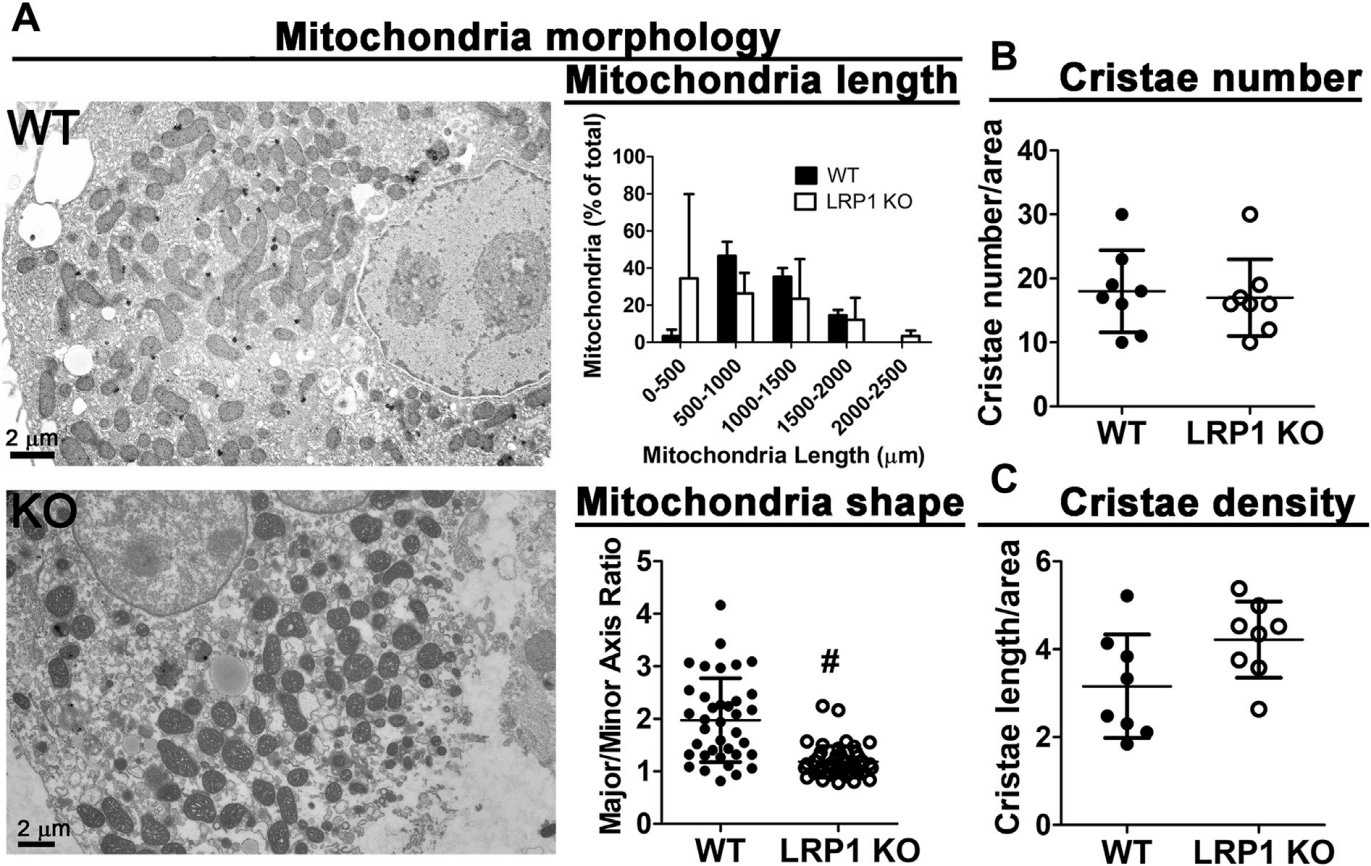
**Hepatic LRP1 deficiency alters mitochondria morphology.** Liver sections from wild-type (WT) and *hLrp1*^*−/−*^ (LRP1 KO) mice were subjected to electron microscopy analysis of the mitochondria. *A*, shows representative images of the liver sections from WT and KO mice. Mitochondria length and shape were determined by measuring ∼40 mitochondria from each mouse (N = 3 per group) and then averaged. *B*, cristae number and *C*, cristae density were determined from the electron microscopy images. The data are reported as mean ± SD (N = 8) and were evaluated by two-tailed Student’s *t*-test for statistical significance. # indicates difference from wild type at *p* < 0.01.

Both respiratory complexes I and II are present in the inner membrane of the mitochondria, and their activities may be regulated by phospholipid composition of the mitochondrial membrane (25). Analysis of phospholipid composition of mitochondria from wild-type and *hLrp1*^*−/−*^ mouse livers by thin layer chromatography revealed reduced levels of anionic phospholipids including phosphatidylethanolamine, cardiolipin, phosphatidylinositol, and phosphatidylserine in the mitochondria of *hLrp1*^*−/−*^ mice compared with wild type (Fig. 5). Since phospholipid composition in the mitochondrial membrane also influences mitochondria morphology (25), we examined the livers isolated from wild-type and *hLrp1*^*−/−*^ mice by transmission electron microscopy and observed significant differences in mitochondrial morphology. The mitochondria in the livers of *hLrp1*^*−/−*^ mice appeared to be rounder than the typical elongated-shape mitochondria observed in wild-type mouse livers (Fig. 6*A*). Despite these differences in shape, no difference in the number of cristae or cristae density was observed between mitochondria from wild-type and *hLrp1*^*−/−*^ mice (Fig. 6, *B* and *C*). These results indicate that LRP1 deficiency alters mitochondrial morphology and activities independent of cristae structure, but is a result of changes in phospholipid composition, particularly the lower phosphatidylethanolamine and cardiolipin contents in the mitochondrial membrane (26, 27, 28, 29).

### LRP1 deficiency promotes mitochondrial fission and inhibits mitochondrial fusion

**Figure 7:**
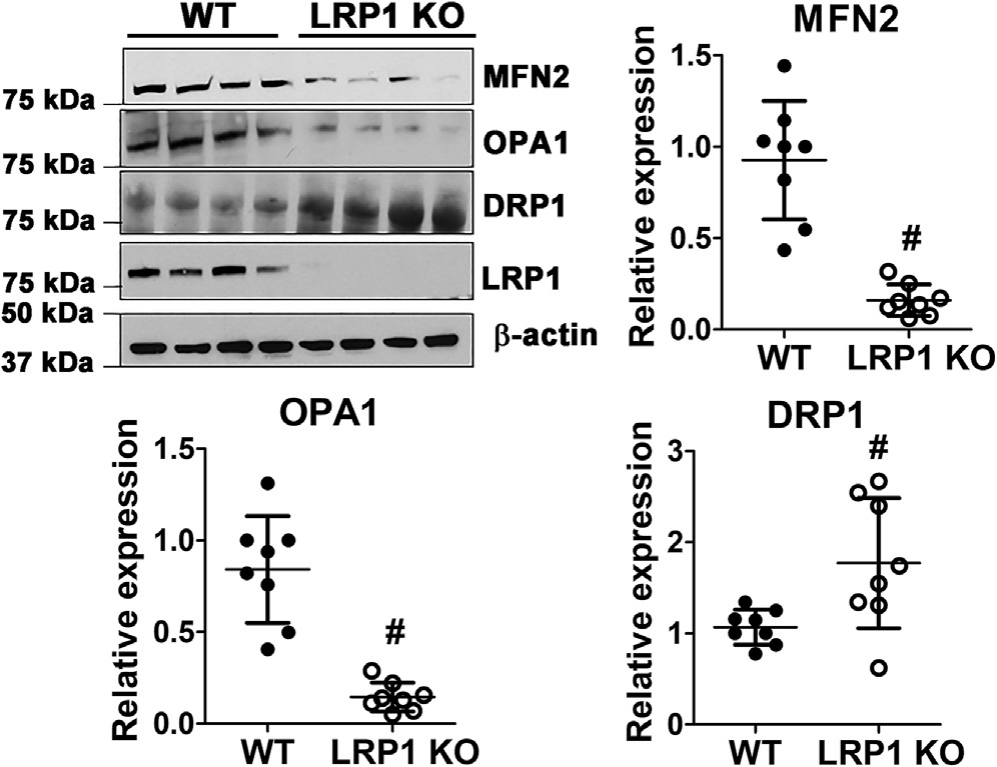
**Hepatic LRP1 deficiency promotes mitochondrial fission and inhibits mitochondrial fusion in the liver.** Liver homogenates from wild-type (WT, *filled symbols*) and *hLrp1*^*−/−*^ (LRP1 KO, *open symbols*) mice (N = 8) were prepared for western blot analysis of MFN2, OPA1, DRP1, and LRP1 expression levels using β-actin levels as the loading control. The images were digitalized by scanning, and quantitative measurements were performed using Image J software. Expression levels of the mitochondrial proteins were normalized to wild-type levels. # indicates statistically significant differences from wild type at *p* ≤ 0.01.

Both cardiolipin and phosphatidylethanolamine appear to play important roles in mitochondrial fusion, potentially through interaction with the inner membrane fusion protein dynamin-like GTPase OPA1 (30). Therefore, we also examined the levels of mitochondrial fusion and fission proteins in the livers of wild-type and *hLrp1*^*−/−*^ mice. Western blot results revealed significant reduction in the levels of the mitochondrial fusion proteins mitofusin-2 (MFN2) and OPA1 in the livers of *hLrp1*^*−/−*^ mice (Fig. 7). In contrast, levels of the dynamin-related protein-1 (DRP1) that is responsible for mitochondrial fission were found to be significantly higher in *hLrp1*^*−/−*^ livers (Fig. 7). These results suggest that the impaired mitochondrial respiration activities observed in *hLrp1*^*−/−*^ hepatocytes may be due to increased mitochondrial fragmentation/fission and the concomitant decrease in mitochondrial fusion.

### LRP1 deficiency reduces phosphatidylinositol(4,5)bisphosphate levels through the reduction of phosphatidylinositol 4-phosphate 5-kinase

**Figure 8:**
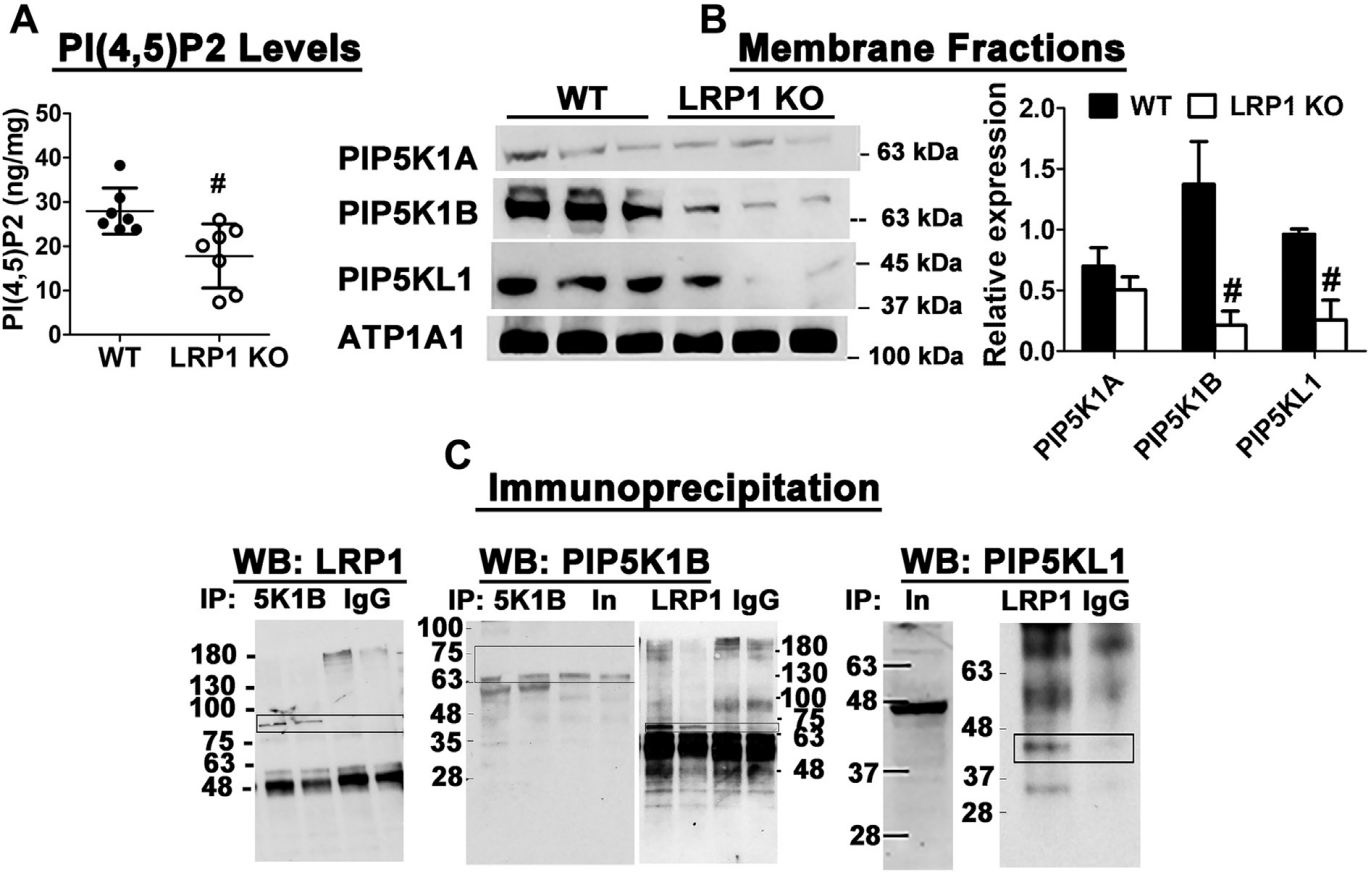
**Hepatic LRP1 deficiency reduces PI(4,5)P**_**2**_**levels by reducing the levels of PIP5K1B and PIP5KL1.***A*, PI(4,5)P_2_ levels in liver extracts obtained from WT and LRP1 KO mice (N = 7) were determined by ELISA. The data were evaluated for statistical significance by two-tailed Student’s *t*-test. # indicates differences from WT group at *p* < 0.01. *B*, liver membrane fractions isolated from WT and LRP1 KO mice were subjected to western blot analysis for expression of PIP5K1A, PIP5K1B, and PIP5KL1 using ATP1A1 as loading control. The images were digitalized and quantified by Image J. # indicates differences at *p* < 0.05. *C*, liver lysates from wild-type mice were subjected to immunoprecipitation (IP) with antibodies against LRP1 or PIP5K1B, using nonspecific IgG as a negative control. The immunoprecipitates were analyzed by western blot (WB) analysis of LRP1, PIP5K1B, and PIP5KL1 as indicated. In, input used for immunoprecipitation. The bands identified as LRP1, PIP5K1B, and PIP5KL1 are outlined in the *box*.

Additional experiments were performed to identify the mechanism by which LRP1 deficiency alters mitochondrial lipid composition and promotes mitochondrial fragmentation. Since the depletion of phosphatidylinositol(4,5)bisphosphate (PI(4,5)P_2_) may increase mitochondrial fragmentation (31, 32), we tested the possibility that LRP1 inactivation may lead to PI(4,5)P_2_ depletion in hepatocytes. Direct ELISA measurement of PI(4,5)P_2_ levels in the livers of wild-type and *hLrp1*^*−/−*^ mice revealed lower PI(4,5)P_2_ levels with LRP1 deficiency (Fig. 8*A*).

Previous studies using proteomics and yeast two-hybrid approaches revealed the potential of LRP1 interaction with phospholipase C-γ1 (PLCγ1) that hydrolyzes PI(4,5)P_2_ to diacylglycerol and inositol trisphosphate (33) and phosphatidylinositol 4-phosphate 5-kinases (PIP5K) and their homologs that synthesize PI(4,5)P_2_ from phosphatidylinositol 4-phosphate (34). Interestingly, whereas the liver membranes from wild-type and *hLrp1*^*−/−*^ mice displayed similar levels of PIP5K1A (Fig. 8*B*), with undetected levels of PLCγ1 and phospho-PLCγ1 (data not shown), significantly lower levels of PIP5K1B and the PIP5K homolog PIP5KL1 were observed in the hepatic membrane fractions of *hLrp1*^*−/−*^ mice compared with wild-type mice (Fig. 8*B*). Additional co-immunoprecipitation experiments showed that LRP1 interacts not only with phosphatidylinositol-4-phosphate 5-kinase-like 1 (PIP5KL1) (Fig. 8*C*), as predicted from the yeast 2-hybrid study (34), but also with PIP5K1B (Fig. 8*C*). Complex between LRP1 and PIP5K1B was confirmed based on pull-down assays with either LRP1 or PIP5K1B antibodies (Fig. 8*D*). Taken together, we interpret these data to indicate that LRP1 binding to PIP5KL1 recruits PIP5K1B to the plasma membrane where it catalyzes PI(4,5)P_2_ synthesis, and the absence of LRP1 reduces PI(4,5)P_2_ levels to enhance mitochondria fission.

## Discussion

This study documents the importance of LRP1 for mitochondria homeostasis in the liver. The absence of LRP1 compromises mitochondrial fusion and enhances mitochondrial fission, resulting in increased mitochondrial fragmentation and functional defects that reduce respiration capacity for ATP production. The mitochondria in the livers of *hLrp1*^*−/−*^ mice also display an abnormal morphology and contain less anionic phospholipids, thus indicating that the functional defects may be due to structural changes caused by the absence of LRP1. In particular, mitochondria in the livers of *hLrp1*^*−/−*^ mice are relatively poor in phosphatidylethanolamine in comparison with the mitochondria in wild-type mouse livers. This anionic phospholipid is predominantly located in mitochondrial outer membrane and is important for appropriate mitochondrial fusion. Mitochondria with phosphatidylethanolamine deficiency are defective in undergoing fusion events due to incomplete mixing of the mitochondrial membranes, thereby increasing fragmentation and mitochondrial fission events (30).

Mitochondria in the livers of *hLrp1*^*−/−*^ mice are also poor in cardiolipin, the anionic phospholipid that is critical for fusion of the inner membrane *via* its interaction and assembly of the dynamin-related GTPase OPA1 (35). Cardiolipin is also present in the outer membrane of the mitochondria where it interacts with another dynamin-related GTPase, DRP1, to mediate mitochondria division and fission events (36). The reduced levels of MFN2 and OPA1 along with the increased levels of DRP1 observed in *hLrp1*^*−/−*^ hepatocytes suggest that LRP1 regulates cardiolipin distribution among inner and outer membranes of the mitochondria, and its deficiency not only reduces cardiolipin levels but also lowers its interaction with the fusion protein OPA1 while preserving its interaction with DRP1 to promote mitochondrial fragmentation and fission. Finally, in addition to the regulation of mitochondria fusion and fission, cardiolipin also plays an important role in mitochondrial bioenergetic processes *via* its interaction and assembly of the electron transport chain complexes involved in oxidative phosphorylation (37). Hence, the lower respiration capacity observed in *hLrp1*^*−/−*^ hepatocytes may also be due to the reduced cardiolipin levels observed in the mitochondrial membrane.

Cardiolipin is synthesized in the mitochondria through cardiolipin synthase-catalyzed condensation of phosphatidylglycerol and cytidine-5’ diphosphate-1,2-diacylglycerol (CDP-DAG) (38), which in turn are generated in the plasma membrane and endoplasmic reticulum through multiple steps involving phosphatidic acid, CTP, diacylglycerol (DAG) kinase, and CDP-DAG synthases. The CDP-DAG present in the endoplasmic reticulum can also be combined with inositol to synthesize phosphatidylinositol by the enzyme phosphatidylinositol synthase. Hence levels of cardiolipin and other anionic phospholipids in cells are regulated by the respective activities of these lipid kinases and synthases. Another determinant of cardiolipin and anionic phospholipid levels in the mitochondria is the availability of substrates including diacylglycerol and phosphatidic acid. In support of the latter possibility is the report showing that activation of PLC, an enzyme that catalyzes DAG and inositol formation from PI(4,5)P_2_, increases CDP-DAG levels in cardiomyoblasts (39). Phospholipase C activation or the addition of DAG mimetic also leads to inhibition of phosphatidylglycerol synthesis to attenuate cardiolipin biosynthesis (40). The current study revealed significantly lower PI(4,5)P_2_ levels in the livers and isolated hepatocytes from *hLrp1*^*−/−*^ mice. Thus the reduced cardiolipin levels observed in the mitochondria of *hLrp1*^*−/−*^ mice may be related to reduced level of PI(4,5)P_2_ substrate for diacylglycerol and cardiolipin synthesis.

**Figure 9:**
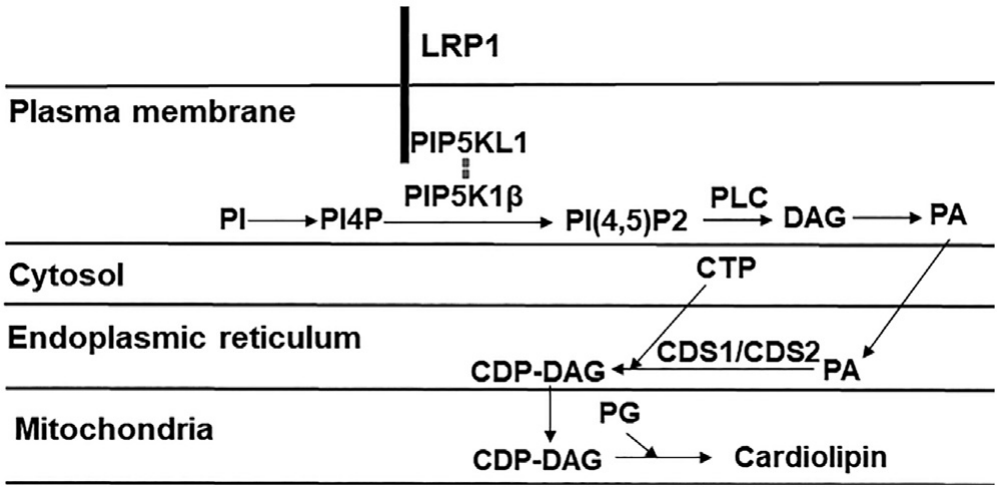
**Schematic diagram depicting the mechanism underlying the role of LRP1 in PI(4,5)P**_**2**_**and cardiolipin synthesis.** The diagram illustrates LRP1, located at the plasma membrane, binds PIP5KL1, and recruits PIP5K1β to the membrane to catalyze PI(4,5)P_2_ synthesis from phosphatidylinositol (PI) and phosphatidylinositol-4-phosphate (PI4). The PI(4,5)P_2_ is hydrolyzed to diacylglycerol (DAG) by phospholipase C (PLC) and then converted to phosphatidic acid (PA), which can be translocated to the endoplasmic reticulum for synthesis of CDP-DAG in the presence of CDP-DAG synthase-1 and -2 (CDS1/CDS2). The CDP-DAG can be translocated from the endoplasmic reticulum to the mitochondria where it can be combined with phosphatidylglycerol (PG) for cardiolipin synthesis.

Intracellular PI(4,5)P_2_ level is exquisitely regulated by its synthesis through the consecutive action of phosphatidylinositol 4-kinase and the phosphatidylinositol 4-phosphate 5-kinases and its utilization by phospholipase C. A previous proteomics study reported the interaction between LRP1 and phospholipase C (33). However, we did not detect any evidence of LRP1-PLCγ1 interaction or aberrant PLCγ1 activation in the livers of *hLrp1*^*−/−*^ mice under basal chow-fed conditions. Although this study cannot rule out the possibility of LRP1-PLCγ1 interaction and aberrant PLCγ1 activation with LRP1 deficiency under stressed conditions, the reduced levels of PI(4,5)P_2_ and cardiolipin observed in chow-fed *hLrp1*^*−/−*^ mice are not due to PLC-mediated PI(4,5)P_2_ utilization. In contrast, our data indicate that LRP1 complexes with PIP5K1β and PIP5KL1 in the liver and LRP1 inactivation reduces the levels of these proteins in the plasma membrane. These results are consistent with a previous yeast 2-hybrid study revealing the potential interaction between LRP1 and PIP5KL1 (34). This study provided documentation of LRP1 and PIP5KL1 interaction in a biological context. Importantly, our results also revealed the importance of this interaction in maintaining PI(4,5)P_2_ levels in the liver. It is interesting to note that PIP5KL1 lacks lipid kinase activity but associates with type 1 PIP kinases such as PIP5K1α or PIP5K1β to generate PI(4,5)P_2_ in specific intracellular compartments (41). Taken together, our data indicate that in hepatocytes, LRP1 interacts with PIP5KL1 and recruits PIP5K1β to the plasma membrane for PI(4,5)P_2_ biosynthesis. The lack of LRP1 results in the impairment of PI(4,5)P_2_ synthesis, thereby reducing the availability of precursor substrates for cardiolipin synthesis and causing mitochondrial dysfunction. A schematic diagram depicting how LRP1 participates in PI(4,5)P_2_ maintenance and cardiolipin biosynthesis in hepatocytes is shown in Figure 9.

Phosphatidylinositol 4,5-bisphosphate generated at the plasma membrane is a key lipid messenger that regulates numerous subcellular processes including endocytosis, exocytosis, endolysosomal trafficking, endosomal recycling, and autophagosomal pathways (32). In this regard, we have shown previously that LRP1 deficiency increases palmitate-induced lipid accumulation and accelerates hepatic cell death. These effects are not the result of defective autolysosomal formation but related to increased lysosome and mitochondria permeability (23). Results of the current study suggest that the increased sensitivity of LRP1-deficient hepatocytes to palmitate-induced mitochondria permeability may be due to the altered mitochondrial membrane composition and fragmentation as a consequence of limited PI(4,5)P_2_ availability for cardiolipin synthesis. In view of the importance of PI(4,5)P_2_ in autophagic lysosome reformation (42), defective lysosome reformation accelerates lysosomal membrane permeability and cell death (43), the lysosomal dysfunction observed in *hLrp1*^*−/−*^ hepatocytes may also be due to the reduced level of PI(4,5)P_2_ in these cells. Taken together, the current study identifies another signaling mechanism by which LRP1 regulates cell functions, *via* binding and recruitment of PIP5KL1 and PIP5K1β to the membrane for PI(4,5)P_2_ synthesis, and highlights the importance of this mechanism for maintaining the integrity and functions of intracellular organelles.

## Experimental procedures

### Materials

Key reagents and resources, including source of antibodies, reagent kits, and primer sequence information, are listed in Table S1 in the Supplement.

### Animals and diets

Hepatocyte-specific LRP1-null (*hLrp1*^*−/−*^) mice were generated by mating *Lrp1*^*flox/flox*^ mice on a C57BL/6J background with albumin promoter-driven recombinase transgenic mice on C57BL/6J background (Jackson Laboratories) as described (20, 22, 23). Male littermates of *hLrp1*^*flox/flox*^ mice without the *cre* transgene were used as wild-type controls. All animals were maintained under controlled environmental conditions with free access to food and water. The mice were anesthetized by isoflurane inhalation prior to tissue harvesting. All experimental procedures and animal care techniques were performed with protocols approved by the University of Cincinnati Institutional Animal Use and Care Committee.

### Primary hepatocytes isolation and culture

Primary hepatocytes were isolated from 20-week-old wild-type and *hLrp1*^*−/−*^ mice after anesthetization by perfusion with Krebs–Henseleit buffer containing 0.5 mM EGTA followed by a digestion solution of Krebs–Henseleit buffer containing 125 units/ml collagenase (Sigma) and 2% bovine serum albumin at a rate of 6 ml/min/g of liver. The hepatocytes were washed in William’s E media (Life Technologies) containing 100 units/ml penicillin, 100 μg/ml streptomycin, and 2 mM L-glutamine, followed by a sedimentation in Percoll solution (GE Healthcare) diluted in phosphate-buffered saline. The cells were incubated overnight in William’s E medium before experiments.

### Cellular oxygen consumption rate

Oxygen consumption rate (OCR) was measured in intact hepatocytes by Seahorse XF24 Extracellular Flux Analyzer as described previously (44). Briefly, primary hepatocytes (10,000 cells/well) in collagen-coated XF24 plates were incubated for 1 h at ambient temperature and then overnight in William’s E medium supplemented with 100 U/ml penicillin/Streptomycin, 2 mM L-glutamine, 5% FBS, 100 nM insulin, and 100 nM dexamethasone. The cells were rinsed twice with PBS and kept at 37 °C in 500 μl of DMEM medium supplemented with 25 mM glucose, 1 mM sodium pyruvate, and 4 mM Glutamax for 1 h in CO_2_-free incubator. Baseline measurements were made to determine basal respiration prior to the sequential delivery of 12 μg/ml oligomycin, 1.5 μM FCCP, and 1 μM rotenone/1.5 μM antimycin A through the instrument’s individual injection ports for the measurements of ATP production and proton leak, maximum respiration, and spare respiration capacity, respectively. In selective experiments, pyruvate plus malate or succinate was used instead of glucose and pyruvate to determine mitochondria complex I- and complex II-specific OCRs.

### Mitochondria isolation

Livers dissected from the animals were immediately placed on ice, diced into <1 mm pieces, and then homogenized in isolation medium (220 mM d-mannitol, 70 mM sucrose, 1 mM EDTA, 10 mM MOPS, 0.5% BSA, pH to 7.2 with KOH). The homogenates were centrifuged at 500*g* for 15 min. The supernatant was strained through gauze and then centrifuged again at 10,000*g* for 15 min to produce a mitochondria pellet. The pellet was resuspended two times in wash medium (250 mM sucrose, 10 mM MOPS, pH to 7.2 with KOH) and recovered by centrifugation at 10,000*g*. The final pellet was resuspended in 250 to 500 μl of wash medium and stored on ice until use. Mitochondrial protein concentration was determined by bicinchoninic acid assay (BCA, Thermo Scientific). Mitochondria number was estimated as described previously based on the ratio of mitochondrial NADH dehydrogenase 1 copy number to nuclear hexokinase 2 copy number determined by PCR using primers indicated in Supplemental Materials (44).

### Mitochondrial calcium uptake

Mitochondrial calcium uptake was determined as described previously (45). Briefly, freshly isolated mitochondria (0.7 mg protein/ml) were incubated in assay medium (250 mM sucrose, 2.5 mM MgCl_2_, 0.5 mM EDTA, 10 mM MOPS, 0.72 mM K_2_HPO_4_, 0.28 mM KH_2_PO_4_, and pH to 7.2 with KOH) supplemented with 5 mM glutamate/5 mM malate as respiratory substrates. For determination of calcium uptake, 1 μM of the Ca^2+^-binding fluorescent probe Calcium Green-5N (Invitrogen) was added to the assay media prior to incubation with 75 μM CaCl_2_. Fluorescent intensity was determined at 538 nm after excitation at 485 nm before and every 2.5 s after CaCl_2_ addition.

### Mitochondrial membrane potential measurement

Safranin O was used as indicator of mitochondrial voltage for estimation of mitochondrial membrane potential (46). The isolated mitochondria were incubated in assay medium containing 25 μM safranin O (Sigma) for 2 min at room temperature prior to the addition of 5 mM succinate. Absorbance at 530 nm was measured prior to and every 30 s after succinate addition to determine membrane potential.

### Mitochondrial morphology determination

The size and shape of mitochondria were determined by electron microscopy. Liver tissues from wild-type and *hLrp1*^*−/−*^ mice were fixed in 3% buffered glutaraldehyde and subjected to ultrathin sectioning and examination with a Zeiss 912 transmission electron microscope as described (47).

### Mitochondria lipid determination

Lipids were extracted from the mitochondria by chloroform:methanol:water (2:1:3, v/v/v) and then dried under nitrogen. The samples were redissolved in chloroform and then applied to thin layer chromatography plates for phospholipid separation. Migration of the phospholipids was compared with standards. Phospholipid spots on the chromatography plates were scraped and quantified by phosphorus measurements.

### Western blot analysis

Hepatocyte cell lysates, liver homogenates, or liver plasma membranes isolated using the procedure as described (48) were incubated with ice-cold radioimmune precipitation assay buffer containing protease and phosphatase inhibitor mixture (Roche Diagnostics). Proteins in the cell lysate were resolved by SDS–polyacrylamide gel electrophoresis and then transferred to polyvinylidene difluoride membranes (BioRad). The membranes were blocked in 5% milk solution containing 0.1% Tween 20 for 1 h at 4 °C and then incubated overnight with a 1:1000 dilution of primary antibodies. The membranes were washed and then incubated with horseradish-peroxidase-conjugated secondary antibodies. The blots were visualized by enhanced chemiluminescence reagents (Pierce). The images were digitalized by scanning, and quantitative measurements were performed using Image J software (National Institutes of Health). Antibodies used in this study were obtained commercially as listed in Table S1.

### Phosphatidylinositol 4,5-bisphosphate measurements

The levels of PI(4,5)P_2_ in hepatocyte lysates were quantified by PIP2 ELISA Kit (MyBiosource) with a calibration curve of 0 to 64 ng/ml PI(4,5)P_2_ according to instructions from the manufacturer.

### Co-immunoprecipitation

Liver protein lysates were precleared with off-target antibody for 1 h at 4 °C and subsequently incubated with Sepharose beads for 20 min at 4 °C. Before adding the precleared protein lysate, antibodies (LRP1 and PIP5K1B) were cross-linked to Protein-A Dynabeads using BS3 cross-linker and incubated with antibody-Dynabead complex overnight at 4 °C on a rotator. Dynabead antibody–lysate complex was washed exhaustively with PBS, prior to eluting the immune-captured proteins with 2X SDS buffer for western blot analysis.

### Statistical analysis

Statistical analysis was performed using SigmaPlot version 13.0 software. Normality was examined using the Shipiro–Wilk test. Data with equal variance based on Brown–Forsythe analysis were evaluated by Student’s *t*-test for studies comparing two groups. Data with unequal variance were evaluated using the Mann–Whitney test. Differences at *p* < 0.05 were considered statistically significant.

## Data availability

The data supporting this study are available in the article and are available from the corresponding author (huidy@ucmail.uc.edu) upon request.

## Supporting information

This article contains supporting information.

## Conflict of interest

The authors declare that they have no conflicts of interest with the contents of this article.
