# Supplementary material for: Hepatic LDL receptor-related protein-1 deficiency alters mitochondrial dynamics through phosphatidylinositol 4,5-bisphosphate reduction
Source: J Biol Chem. 2021 Feb 3;296:100370. doi: 10.1016/j.jbc.2021.100370 (PMC7949165; doi:10.1016/j.jbc.2021.100370)
Supplement: Supplemental Table S1 [file mmc1.pdf]

SUPPORTING INFORMATION

MATERIALS

Table S1. Key Reagents.

REAGENTS or RESOURCES	SOURCE	IDENTIFIER
**Antibodies**		
MFN2	Cell Signaling	9428S
OPA1	Novus Biologicals	NB110-55290
DRP1	Novus Biologicals	NB110-55237
$PI(4,5)P_2$	Abcam	Ab11039
β-actin	Cell Signaling	4970S
PIP5K1A	Protein Tech	15713-1-AP
PIP5K1B	Protein Tech	12541-1-AP
PI5KL1	Protein Tech	17547-1-AP
PLCγ1	Cell Signaling	2822S
HRP-conjugated antibody	Cell Signaling	7074
Alexa Fluor® 594	Life Technologies	A21203
Total OXPHOS WB antibody cocktail	Abcam	Ab110413
**Commercial Assay Kits**		
$PI(4,5)P_2$	MyBioSource	MBS1604532
**Key Reagents, Materials**		
Glutamax	Invitrogen	35050-061
Oligomycin	Sigma-Aldrich Chemicals	O4876-5MG
FCCP	Sigma-Aldrich Chemicals	C2920-10MG
Rotenone	Sigma-Aldrich Chemicals	R8875-1G
Antimycin A	Sigma-Aldrich Chemicals	A8674-25MG
Safranin O	Sigma-Aldrich Chemicals	S2255
Calcium Green – 5N	Invitrogen/Thermo Fisher	C3737
RIPA Lysis and Extraction Buffer	Thermo Fisher Scientific	89900
BS3 crosslinker	Thermo Fisher	21580
Protein A-Sepharose	Thermo Fisher	10-1041
Protein A-Dynabeads	Life Technologies	10001D
PhosphoStop Phosphatase Inhibitor	Roche Applied Science	4906837001
Protease Inhibitor	Roche Applied Bioscience	11697498001
ExpressPlus PAGE Gels	GenScript	M41210
Li-Cor Odyssey Blocking Buffer	Li-Cor Biosciences	927-40000
Pierce ECL Western Blot Substrate	Thermo Fisher	PI32106
Direct-zol RNA MiniPrep	Zymo Research	R2052
qScript cDNA Synthesis Kits	VWR	101414-098
Fast SYBR Green Master Mix	Life Technologies	4385612
**Primer Sequences for PCR**		
**Gene**	**Forward Primer**	**Reverse Primer**
ND	CTAGCAGAAACAAACCGGGC	CCGGCTGCGTATTCTACGTT
HK	GCCAGCCTCTCCTGATTTTAGTGT	GGGAACACAAAAGACCTCTTCTGG
